# CD47 expression in non-small cell lung cancer and its relationship with tumor-associated macrophage infiltration

**DOI:** 10.1371/journal.pone.0314228

**Published:** 2024-12-09

**Authors:** Hefeng Zhang, Shihu Liu, Jinzi Zhang, Yongjie Wang

**Affiliations:** Department of Thoracic Surgery, The Affiliated Hospital of Qingdao University, Qingdao, Shandong, China; City of Hope National Medical Center, UNITED STATES OF AMERICA

## Abstract

**Background:**

Non-small cell lung cancer (NSCLC) has a high incidence, with most patients diagnosed beyond the optimal surgical window. Improving survival rates is critical to reducing lung cancer mortality, and identifying immune checkpoints is vital for prognosis stratification.

**Objective:**

To investigate the expression of CD47 in NSCLC and its relationship with tumor-associated macrophage infiltration.

**Methods:**

A retrospective analysis was conducted on 50 NSCLC patients treated between January 2014 and June 2018. Immunohistochemistry and confocal microscopy assessed CD47 expression in tumor and adjacent tissues, while immunofluorescence evaluated CD47 on tumor-infiltrating T lymphocytes. Kaplan-Meier survival analysis and Cox regression identified prognostic factors, and PLA technology examined CD47’s interaction with VEGFR and CD36.

**Results:**

CD47 positivity in tumor tissues (52%) was significantly higher than in adjacent tissues (20%) (*P*<0.001), with expression localized to the cell membrane. CD47 expression correlated with lymph node metastasis, clinical stage, and differentiation (*P*<0.05) and was identified as an independent risk factor for poor prognosis. TAM infiltration was greater in CD47-positive patients and correlated with shorter survival (*P*<0.05). PLA showed stronger CD47+VEGFR interactions than CD47+CD36.

**Conclusion:**

CD47 positivity correlates with poor prognosis and increased TAM infiltration, highlighting its potential as a prognostic biomarker and therapeutic target in NSCLC.

## 1. Introduction

Lung cancer is currently one of the cancer types with the highest incidence and mortality rates worldwide, which seriously affects human health and life [1]. According to the recent statistics from the American Cancer Society (ACS), 1,958,310 new cancer cases and 609,820 cancer deaths are expected to occur in the United States in 2023, of which 238,340 new lung cancer cases are predicted, accounting for 12.17% of the total number of new cancer cases. The number of new cases of lung cancer is predicted to be 238,340, accounting for 12.17% of all new cases of cancer, and the number of deaths due to lung cancer in the coming year is estimated to be 127,070, accounting for 20.83% of the total number of cancer deaths. Non-small cell lung cancer (NSCLC) accounts for more than 80% of all lung cancers [2,3]. How to reduce the mortality of NSCLC patients is a problem that cannot be ignored, and effective prognostic indicators are important for the subsequent individualized treatment of patients.

CD47 is a cell surface glycoprotein and belongs to the immunoglobulin superfamily (Ig superfamily) that is ubiquitously expressed on human cells and can bind to a variety of proteins, including integrins,Tumor-associated macrophages primarily originate from monocytes and are differentiated into macrophages through signals from the tumor microenvironment. [4]. In recent years, the role of CD47 for tumor cells and immune cells has made some progress. Tumor cells evade immune attack through high expression of CD47, which leads to proliferation, invasion and metastasis (e.g., gastric cancer, colon cancer, leukemia, etc.) [5–7]. Meanwhile, studies have shown that CD47 can regulate cell migration and phagocytosis as well as immune homeostasis by acting on macrophages, granulocytes, monocytes, and bone marrow dendritic cells [8], thus regulating the immune environment of the body. However, the role of CD47 in the tumor microenvironment of non-small cell lung cancer has still not been exhaustively described, especially the role of CD47 in tumor-associated macrophages in non-small cell lung cancer has not been studied. In breast cancer, Upton [9] et al. demonstrated enhanced phagocytosis of tumor-associated macrophages by blocking the CD47 / SIRP α checkpoint to enable trastuzumab-mediated phagocytosis of HER2 tumor cells by trastuzumab-mediated macrophages, suggesting that the use of CD47 / SIRP α checkpoint to block the combination of trastuzumab with trastuzumab for the treatment of HER2 breast cancer (BC) and other drug-resistant HER2 cancers (i.e., gastric cancer, bladder cancer, etc.) with therapeutic potential. In thyroid cancer, intervention in a mouse model using anti-CD47 antibody revealed increased tumor-associated macrophage infiltration, enhanced phagocytosis of tumor cells and inhibited tumor growth [10]. In lung cancer, the relationship between CD47 and tumor-associated macrophages is unclear, whether CD47 plays a role in affecting the prognosis of NSCLC treatment by influencing macrophage differentiation and biological behavior in the NSCLC tumor microenvironment has not been reported in the literature to date, so this was included as the focus of this study.

## 2. Objects and methods

### 2.1 Study subjects

The clinical data of 50 NSCLC patients who received primary treatment in our hospital from January 2014 to June 2018 were analyzed retrospectively from February 2024 to June 2024 by means of electronic medical record query. Among them, 30 cases were male and 20 cases were female. Inclusion criteria: (1) no neoadjuvant radiotherapy, chemotherapy or other treatments were performed before surgery; (2) primary NSCLC was confirmed by pathological examination after surgery; (3) tumor tissues and paracancerous tissues were preserved with liquid nitrogen frozen specimens; (4) the patients were treated with a platinum-based two-drug combination of a circumferential whole-body intravenous chemotherapy regimen after surgery; and (5) the clinical data and follow-up data were complete. Exclusion criteria: (1) age <18 years old or >80 years old; (2) combined with other tumors; (3) combined with functional disorders or organic lesion diseases; (4) combined with acute infection patients.

### 2.2 Methods

This study, titled “CD47 expression in non-small cell lung cancer and its relationship with tumor-associated macrophage infiltration,” was conducted following ethical guidelines and principles. The research protocol was reviewed and approved by the Medical Ethics Committee of the Affiliated Hospital of Qingdao University (Approval No.: QYFYWZLL.28949).

#### 2.2.1 Clinicopathologic features

The clinicopathological characteristics of all patients were analyzed retrospectively, including age, gender, smoking (smoking was defined in the 2002 China Population Health and Nutrition Survey [11], and smoking was defined as having smoked 100 cigarettes for 6 consecutive or cumulative months or more in a lifetime), and underlying diseases (an underlying disease was defined as having an underlying disease if one or more of the following were present: hypertension, diabetes mellitus, coronary heart disease, and coronary artery disease), BMI, pathological type, degree of differentiation, clinical staging, pathology of lung cancer, and clinical staging with reference to the eighth edition of the International Union Against Cancer (UICC) TNM Lung Cancer Staging.BMI = body mass/height^2^ (kg/m^2^), and its grouping criteria were: BMI<18 kg/m^2^ as underweight, 18 kg/m^2^ ≤BMI<24 kg/m^2^ as normal body mass, and 24 kg/m^2^ ≤ BMI<28 kg/m^2^ as overweight, and BMI≥28 kg/m^2^ as obese [12].

#### 2.2.2 Immunohistochemical analysis of CD47 and CD204 expression

Immunohistochemical quantification of CD47 and CD204 expression was performed in NSCLC lung tissue (First, through pathological examination, identify areas where cancer cells are aggregated. The ROI for cancer tissue should include a clear cluster of cancer cells to ensure representativeness. Select areas with typical tumor morphological characteristics, avoiding regions with necrosis, hemorrhage, or other factors that may interfere with observation.) and adjacent non-cancerous samples (samples obtained from non-cancerous tissues within 2 cm of the tumor margin, ensuring that this area was not directly invaded by the tumor or underwent malignant transformation). These samples were used as controls to compare the differences in CD47 expression between tumor tissues and adjacent non-cancerous tissues.Tissue samples were surgically generated and immediately fixed in neutral buffered formalin 3.7%), followed by standardized paraffin matting. Immunohistochemistry was initiated by dewaxing formalin-fixed paraffin-embedded tissue sections (3 μm) in xylol. Paraffin-embedded tissue sections were heated in a microwave oven with 0.01 mol/L citrate buffer (pH 6.0) for 10 min. after cooling to room temperature, endogenous peroxidase was inactivated by the addition of 3% H2O2 for 10 min, and a blocking solution was applied in order to prevent nonspecific binding of the primary antibody. Tissue slides were incubated consecutively overnight for 16h with the following antibodies: anti-CD204 (Sigma) and anti-CD47 (Sigma), The Sigma antibody used in this study targets the external domain of CD47, a region that plays a critical role in CD47’s function by mediating its interactions with other cellular factors.nd antibody reactivity was obtained using the ZytoChemPlus horseradishperoxidase (HRP) polymer system (mouse/rabbit) (ZytomedSystems). Analysis. Substrate and stain [diaminobenzidine (DAB); Dako was applied to the samples. Restaining was obtained with Mayer’s acid hematoxylin. After dehydration of the slides in elevated rows of ethanol, the slides were covered. The results of image acquisition were analyzed by microscopic observation. Two experienced pathologists were involved in the evaluation by reading the slides independently using a double-blind method. Ten high magnification fields of view were randomly selected to evaluate the staining intensity of the sections and the percentage of positive cells. The sections were divided into cancerous and paracancerous tissues. Positive cells were visualized under the microscope by the presence of brown granules. According to the intensity of staining, the sections were divided into four grades: no staining (0 points), light yellow (1 point), brownish yellow (2 points), and tan (3 points), and the proportion of staining was categorized into 0%-100% according to the percentage of staining-positive cells, and the product of the intensity of staining and the proportion of staining was used as the criterion for the interpretation of the results in this experiment. No staining and light yellow color were regarded as low infiltration, and brownish yellow and tan color were regarded as high infiltration. See Table 1.

**Table 1:** Antibody clones and dilution ratio.

Antibody Name	Catalog Number	Manufacturer	Dilution	Isotype	Company
Anti-CD204	ABC123	XYZ Inc.	1:200	IgG1	Sigma-Aldrich
Anti-CD47	DEF456	XYZ Inc.	1:100	IgG2b	Sigma-Aldrich
In Vitro Factor	GHI789	ABC Corp.	1:50	IgM	ABC Corp.
Anti-VEGFR	2479S	Cell Signaling Technology	1:100	Rabbit IgG	Cell Signaling Technology
Anrti-CD36	NB400144	Novus Biologicals	1:200	Mouse IgG	Novus Biologicals

#### 2.2.3 Immunofluorescence confocal microscopy

(1) Select healthy cells, digest, centrifuge, and resuspend thoroughly to obtain a uniform cell suspension for use.(2) Place appropriately sized cell coverslips into a 12-well plate, add the cell suspension, and incubate so that after 12–16 hours, the cell density on the coverslip reaches approximately 70%.(3) After 12–16 hours, observe the cell status and distribution under a microscope, discard the supernatant, and wash twice with PBS, ensuring complete removal of the supernatant.(4) Add 50 μL of DID membrane dye (red fluorescence, working concentration of approximately 30 μM), prepared according to the manufacturer’s instructions, onto the coverslip, and incubate at 37°C for 20–30 minutes.(5) After staining, wash twice with PBS and discard the supernatant.(6) Add 500 μL of 4% paraformaldehyde fixative, fix at room temperature for 20 minutes, then wash twice with PBS, discarding the supernatant.(7) Add 50 μL of DAPI nuclear dye (blue fluorescence, working concentration of approximately 30 μM), prepared according to the manufacturer’s instructions, onto the coverslip and stain at room temperature for 5–10 minutes.(8) After staining, wash twice with PBS and carefully remove any moisture from the coverslip surface.(9) Prepare a glass slide with appropriate labeling, add 30–50 μL of mounting medium to the center of the slide, and place the coverslip onto the mounting medium, allowing it to air dry to secure the coverslip.(10) Observe and capture images using a confocal microscope, and analyze the images for results. (Note: Perform all steps with light protection.)

#### 2.2.4 PLA technique

Cell Fixation: Culture cells on coverslips, fix with 4% paraformaldehyde for 15 minutes, then wash with PBS.Permeabilization: Treat cells with 0.1–0.5% Triton X-100 for 5–10 minutes to increase antibody penetration, then wash with PBS.Blocking: Incubate with 1% BSA or 10% normal serum (matched to secondary antibody host species) at room temperature for 1 hour to reduce nonspecific binding.Primary Antibody Incubation: Use specific antibodies against CD47 and a target protein (VEGFR or CD36), sourced from different species to ensure probe specificity. Incubate antibodies at the recommended dilution overnight at 4°C, then wash with PBS to remove unbound antibodies.PLA Probe Incubation: Select PLA probes matched to primary antibody species (e.g., mouse anti-CD36, rabbit anti-VEGFR), incubate at 37°C for 1 hour, and wash with buffer to remove unbound probes.Ligation Reaction: Add ligation solution (containing short DNA sequences) and incubate at 37°C for 30 minutes to allow DNA linkage between probes.Amplification Reaction: Add DNA polymerase to initiate rolling-circle amplification, generating detectable signals, and incubate at 37°C for 90 minutes.Fluorescent Signal Detection: Stain nuclei with DAPI or another nuclear dye to identify cell morphology, then observe with a fluorescence microscope. PLA signals appear as fluorescent dots, indicating CD47 interactions with VEGFR or CD36. Data Analysis: Capture images and quantify PLA signals per cell using software (e.g., ImageJ) to evaluate CD47 interaction frequency with VEGFR or CD36.

#### 2.2.5 Immunofluorescence detection of CD47 expression on tumor-infiltrating T cells

Add 100 μL of single-cell suspension from separated tissue to an EP tube, then add 2 μL each of anti-human CD4, CD8, CD47, and PD-1 fluorescent antibodies. Incubate on ice, protected from light, for 30 minutes, wash twice with 1 mL PBS, and resuspend cells in 500 μL PBS. For the control, add 100 μL of cell suspension to a separate EP tube without antibodies, incubate on ice in the dark for 30 minutes, wash twice with 1 mL PBS, and resuspend in 500 μL PBS.

#### 2.2.6 Follow-up

Follow-up is conducted at the end of treatment through outpatient review, telephone or SMS; follow-up includes patients’ general condition and recurrence/progression status, survival outcome, etc.; follow-up frequency is 2–3 months within 2 years, half-yearly within 2–5 years, and annually for more than 5 years after the end of treatment; the endpoint of follow-up is death; the follow-up time is up to 2024-02-01, and OS is defined as the time from the beginning of treatment to death or the last follow-up. 01. OS was defined as the time from the start of patient treatment to death or the last follow-up visit.

### 2.3 Statistical methods

The follow-up cutoff for the cohort was January 30, 2021. We analyzed the prognosis by Kaplan-Meier survival analysis. We determined the effect of different factors on OS in NSCLC patients by Cox univariate/multivariate regression analysis. Correlation was done by Spearman analysis. Statistical analysis of this study were done through SPSS 20.0 analysis software and statistical graphing was done through Graphpad Prism 8.0. In this study, *P<0.05, **P<0.01 were considered statistically significant differences.

## 3. Results

### 3.1 CD47 expression in NSCLC cancer tissues and paracancerous tissues

**Fig 1:**
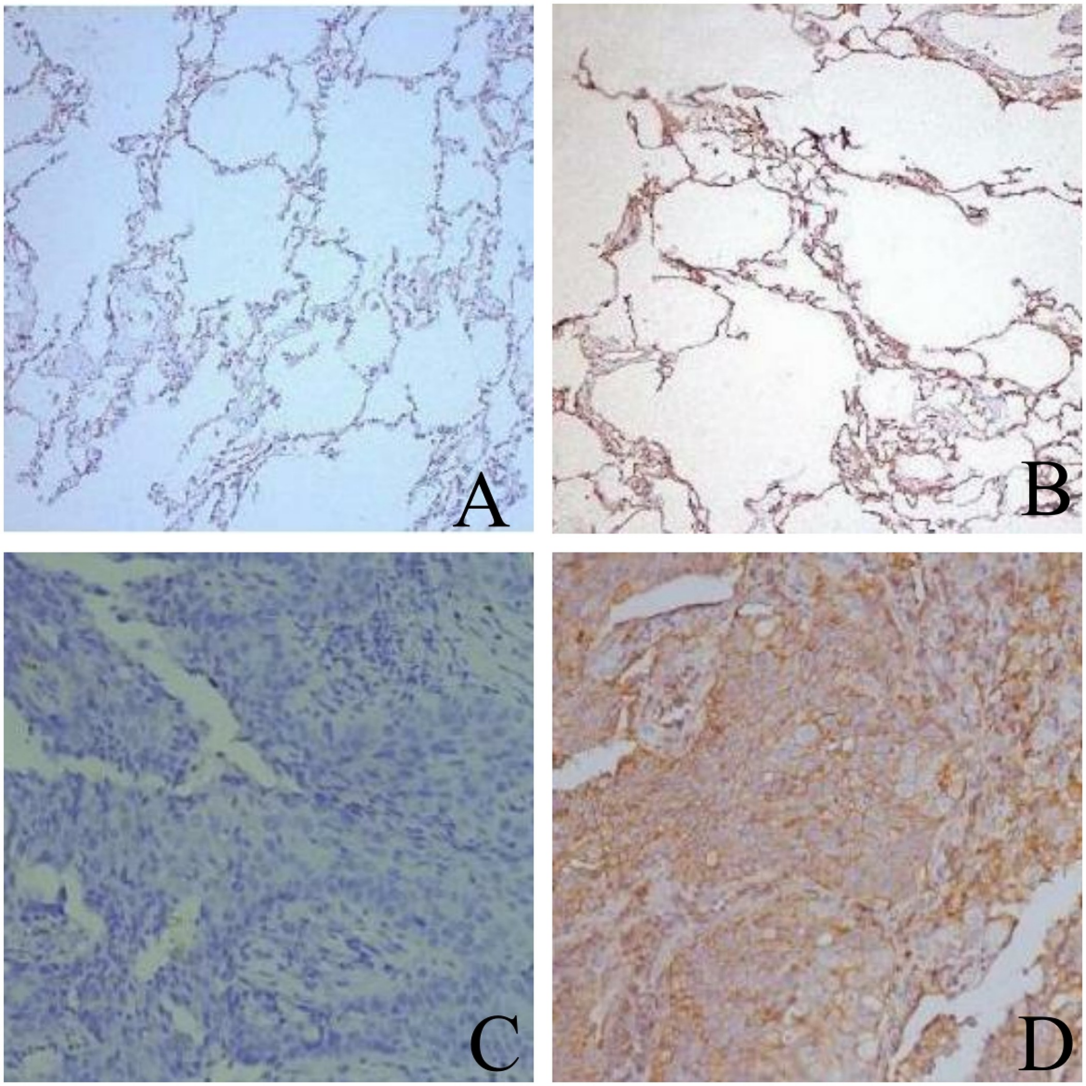
Expression pattern of CD47 in non-small cell lung cancer tissues. High CD47 expression is associated with tumor infiltration. Scale bar = 50 μm. A Paraneoplastic tissue (negative) × 200 B Paraneoplastic tissue (positive) × 200 C Lung cancer tissue (negative) × 200 D Lung cancer tissue (positive) × 200.

Immunohistochemical staining results showed that a large amount of CD47 protein was distributed on NSCLC cancer cells, with a smaller amount present in the cytoplasm. Among the 50 patients, 24 cases exhibited negative expression and 26 cases exhibited positive expression of CD47 in tumor tissues, while in adjacent non-cancerous tissues, 16 cases were negative and 4 cases were positive for CD47 expression. At least five high-power fields (HPFs) were examined for each sample to ensure the reliability of the results. CD47 expression was observed as a strong positive signal on the cell membrane, with some expression also detected in the cytoplasm. The positive expression rate of CD47 in tumor tissues was significantly higher than that in adjacent non-cancerous tissues, with the difference being statistically significant (*P*< 0.05). Among the 24 tumor tissue samples with negative expression, 8 cases also showed negative expression in adjacent tissues, indicating a correlation between the two tissue types (Fig 1).

### 3.2 CD47 is primarily localized on the cell membrane of NSCLC cells

**Fig 2:**
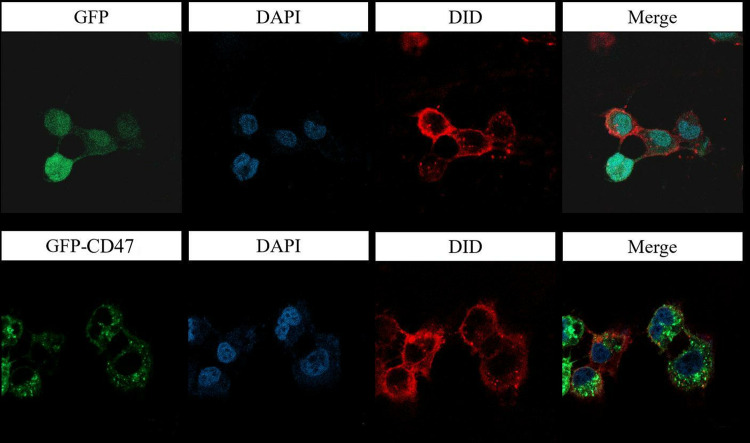
CD47 localization on the cell membrane of NSCLC cells. Scale bar = 10 μm.

We observed the expression and localization of CD47 in NSCLC cells using laser confocal microscopy. The results showed that CD47 is expressed on the cell membrane of NSCLC cells (Fig 2).

### 3.3 Correlation between CD47 protein expression and clinicopathologic features of NSCLC patients

Immunohistochemical staining results showed that a large amount of CD47 protein was distributed on NSCLC cancer cells, with a smaller amount present in the cytoplasm. Among 50 patients, 24 cases exhibited negative expression and 26 cases exhibited positive expression of CD47 in tumor tissues, while in adjacent non-cancerous tissues, 16 cases were negative and 4 cases were positive for CD47 expression. At least five high-power fields (HPFs) were observed for each sample to ensure the reliability of the results. CD47 expression was observed as strong positive signals on the cell membrane, with some expression also detected in the cytoplasm. The positive expression rate of CD47 in tumor tissues was significantly higher than that in adjacent tissues, with the difference being statistically significant (*P*< 0.05). Among the 24 tumor tissue samples with negative expression, 8 cases also showed negative expression in adjacent tissues, indicating a correlation between the two tissue types.as shown in Table 2.

**Table 2:** Correlation between CD47 protein expression and clinicopathologic features of NSCLC patients.

Diagnostic trait	Number of cases	CD47		*χ^2^*(be) worth	P-value
		negatives	masculine		
Age				0.654	0.419
<60 years	20	11	9		
≥ 60 years	30	13	17		
Distinguishing between the sexes					
male	26	11	15	0.703	0.402
female	24	13	11		
Cigarette smoking				1.342	0.247
be	23	9	14		
clogged	27	15	12		
Underlying disease				0.597	0.742
High blood pressure	8	2	6		
Diabetes	7	3	4		
Coronary heart disease	5	2	3		
BMI					
BMI <18kg/m ^2^	20	10	10	0.136	0.934
24 kg/m^2^ ≤BMI<28 kg/m^2^	13	7	6		
BMI ≥28 kg/m^2^	17	8	9		
Pathological characteristics				1.290	0.256
Squamous carcinoma	13	8	5		
Adenocarcinoma	37	16	21		
Degree of differentiation				11.02	0.004
High differentiation	21	1	20		
Middle ground	15	7	8		
Low polarization	14	7	7		
Clinical staging				6.270	0.043
Ⅰ	18	12	6		
II	13	7	6		
III	19	5	14		
Lymphatic node transfer				6.559	0.010
There are	24	7	17		
Not have	26	17	9		

### 3.4 Univariate and multivariate analysis of the effect of each indicator on overall survival

The 50 NSCLC patients were grouped according to the different follow-up data, and the Kaplan-Meier method was utilized to compare the correlation between clinical indexes and OS in each group.The results of Cox unfolding multifactorial analysis showed that CD47 positive expression and clinical stage were the independent risk factors affecting the prognosis, as shown in Tables 3 and 4.

**Table 3:** Univariate analysis of the effect of indicators on overall survival.

Diagnostic trait	Average PFS (months)	Median PFS (months)	*χ^2^*(be) worth	P-value
Age			0.599	0.439
<60 years	34.3	26.2		
≥ 60 years	28.4	16.0		
Distinguishing between the sexes			0.229	0.633
male	33.5	21.8		
women	26.0	26.2		
Smoking history			0.003	0.955
there are	31.3	21.8		
not have	30.1	26.4		
Clinical staging			28.107	0.000
Ⅰ	44.0	34.2		
II	29.3	20.1		
III	15.8	15.4		
Pathological type			1.882	0.169
squamous carcinoma	41.5	21.8		
adenocarcinoma	25.0	25.0		
Degree of differentiation			2.506	0.288
high differentiation	37.5	26.2		
middle ground	29.0	16.0		
low polarization	22.6	24.7		
Lymphatic node transfer			15.992	0.000
there are	19.0	13.1		
not have	42.0	31.6		
CD47 expression			8.551	0.003
negatives	39.8	31.6		
masculine	20.3	14.6		

**Table 4:** Multifactorial analysis of prognosis of NSCLC patients.

diagnostic trait	P-value	B	95% *CI*
clinical staging	0.000	2.095	1.318 to 3.331
lymphatic node transfer	0.482	0.731	0.302∼1.782
CD47 positive expression	0.041	2.282	1.145 to 4.543

### 3.5 Expression levels of tumor-associated macrophages in NSCLC tissues

**Fig 3:**
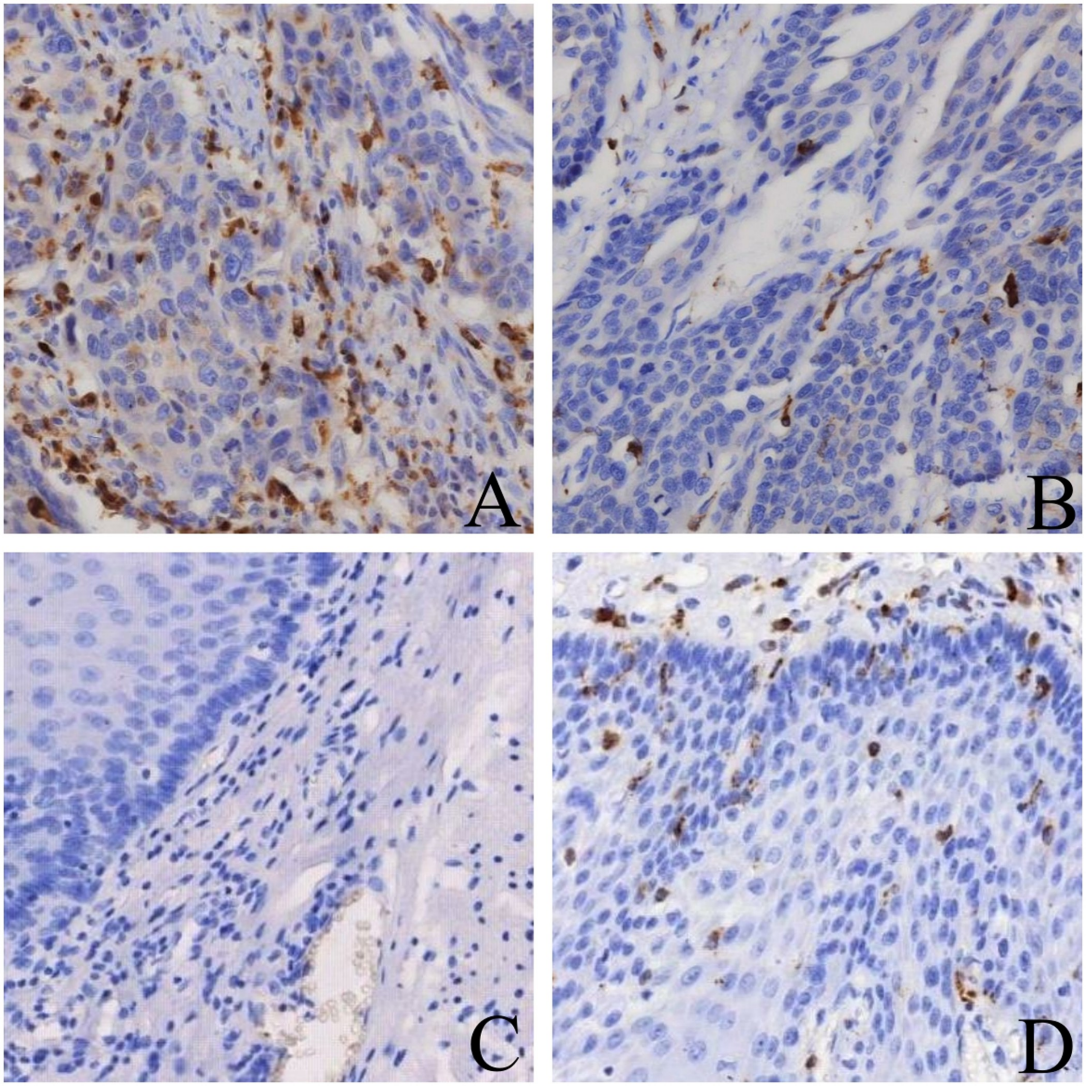
Expression of CD204 in adjacent non-cancerous tissues. The data show low CD204 expression in adjacent non-cancerous tissues. Scale bar = 50 μm. AHigh expression of CD204 in NSCLC (×400) B Low expression of CD204 in NSCLC (×400) CNegative expression of CD204 in paraneoplastic tissue (×400) DHigh expression of CD204 in paraneoplastic tissue (×400).

CD204 is abundantly expressed on the membrane of macrophages, appearing as brown-yellow staining. The expression of CD204 can be evaluated based on the intensity and extent of the staining. CD204 is specifically expressed in M2-type macrophages, and therefore, the distribution of macrophages can be assessed by the presence of CD204-positive cells. For each sample, at least five high-power fields (HPF) were examined to ensure the reliability of the results. In tumor tissues, the median number of CD204-positive tumor-associated macrophages (TAMs) was 35 cells/HPF, whereas in adjacent non-cancerous tissues, the median was 4 cells/HPF. Statistical analysis revealed that the expression level of CD204 in tumor tissues was significantly higher than in adjacent tissues (*P* < 0.001). Among the 24 tumor tissue samples with negative CD204 expression, 7 cases also showed negative expression in adjacent tissues, indicating a certain degree of consistency in CD204 expression between these two tissue types(Fig 3).

### 3.6 Effect of tumor macrophage infiltration on OS in NSCLC lung tissue

**Fig 4:**
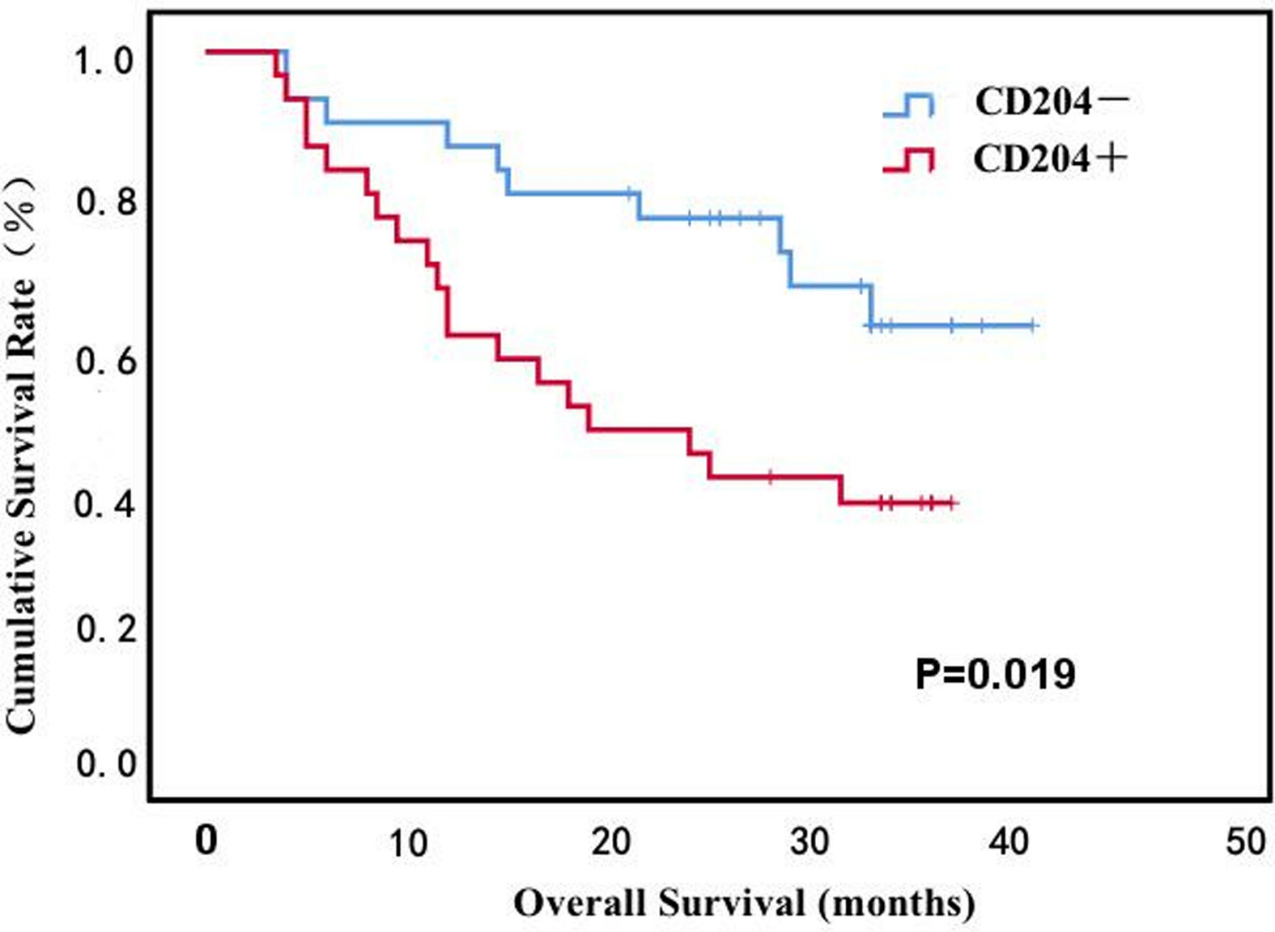
CD204 protein expression Kaplan-Meier survival analysis.

The OS of 50 NSCLC patients was analyzed.The results of the Kaplan-Meier method showed that the difference between tumor macrophage infiltration and patient survival was statistically significant (*P* < 0.05), and the less tumor macrophage infiltration, the longer OS of NSCLC patients (Fig 4).

### 3.7 Correlation between CD47 positive expression and tumor macrophage infiltration in NSCLC patients

**Fig 5:**
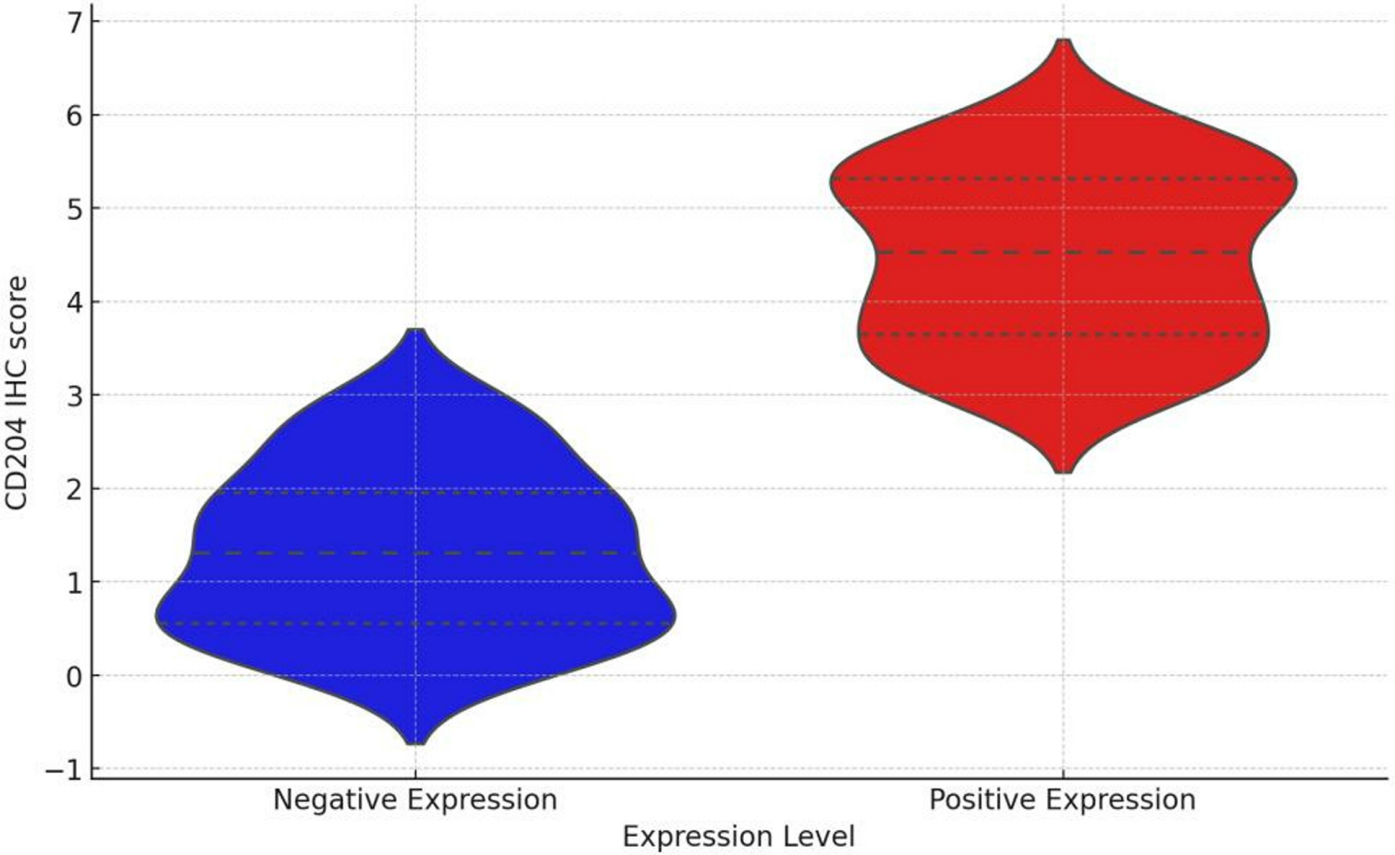
Correlation between CD47 positive expression and tumor macrophage infiltration in NSCLC patients.

Tumor macrophage infiltration was significantly higher in patients with CD47-positive expression than in patients with CD47-negative expression (*P* < 0.05) (Fig 5).

### 3.8 Interaction of CD47 with VEGFR and CD36 detected by PLA

**Fig 6:**
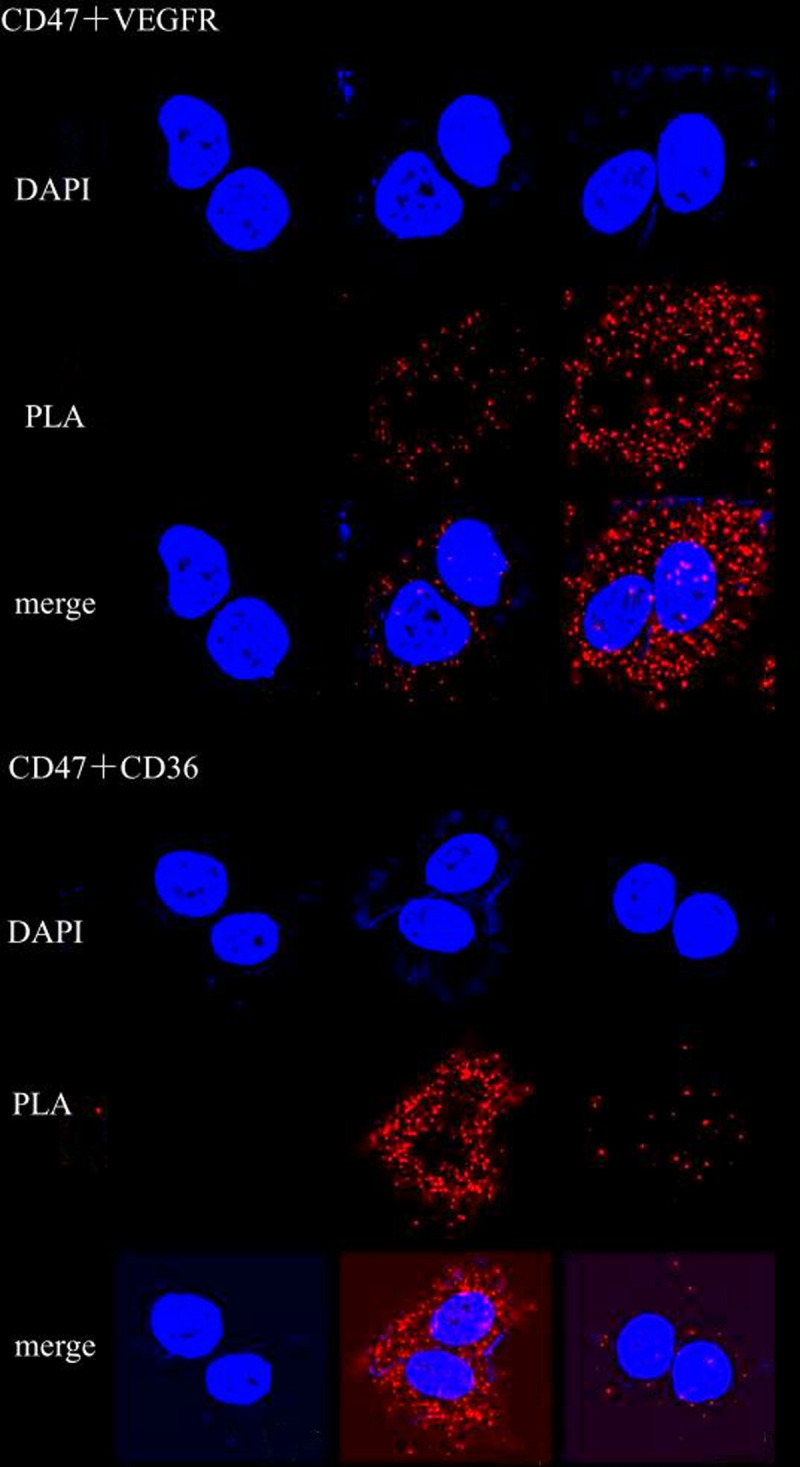
Interaction of CD47 with VEGFR and CD36 detected by PLA. Scale bar = 50 μm.

**Fig 7:**
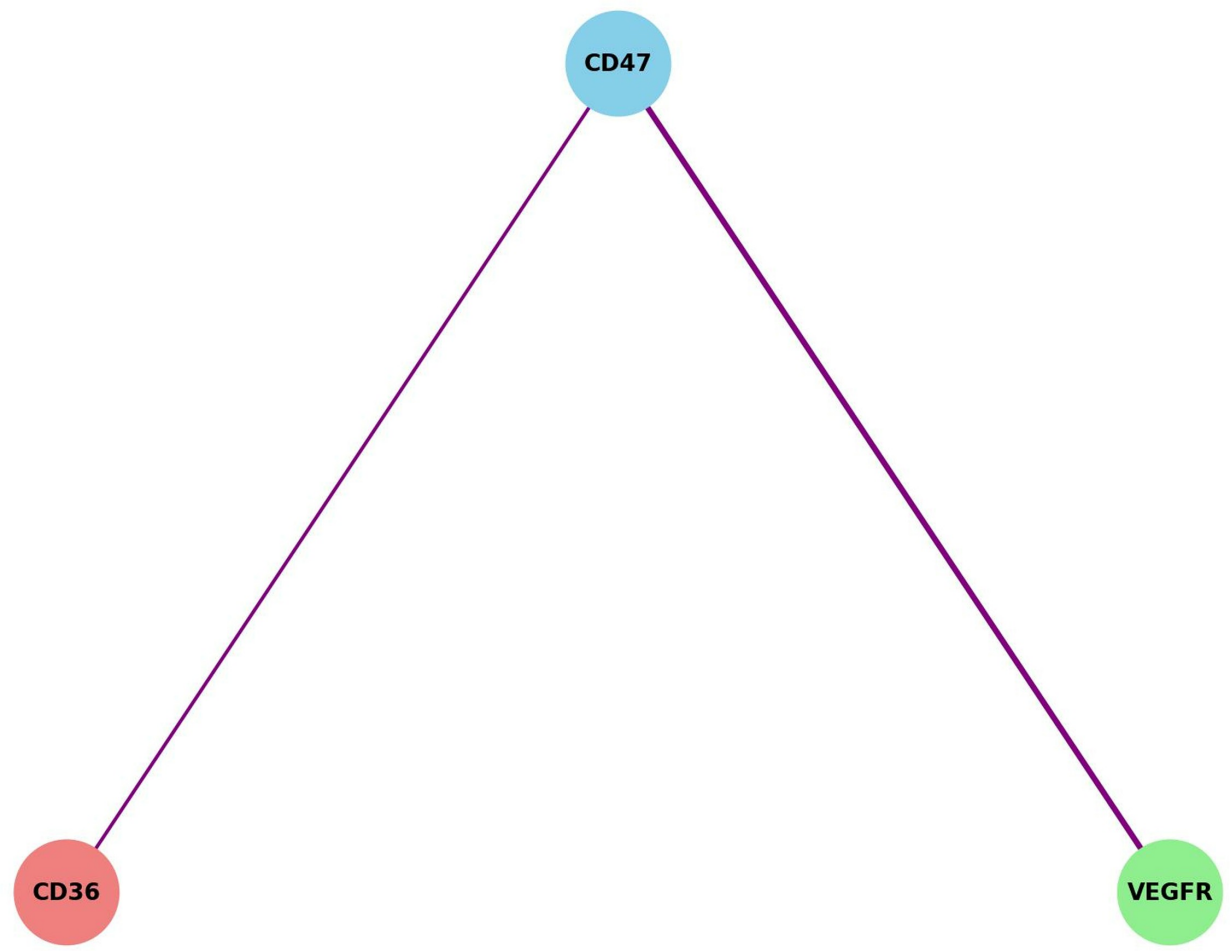
Interaction diagram of CD47 with VEGFR and CD36.

To further confirm the interaction between CD47 and VEGFR or CD36, we used the in situ proximity ligation assay (PLA). This technique relies on a pair of oligonucleotide-labeled antibodies that are closely conjugated (approximately 30–40 nm apart) to different epitopes on the same protein or between two proteins in a complex. By observing the punctate fluorescence signals, we found that PA treatment significantly increased positive fluorescent signals in NSCLC cells compared to the control group. This result aligns with our previous immunofluorescence findings, with the CD47+VEGFR group displaying stronger positive signals than the CD47+CD36 group (Figs 6 and 7).

Annotation: The line connecting CD47 and VEGFR is thicker, indicating a stronger interaction, while the line between CD47 and CD36 is thinner, representing a weaker interaction.

### 3.9 CD47 is expressed at low levels on tumor-infiltrating CD4+ and CD8+ T lymphocytes in NSCLC

**Fig 8:**
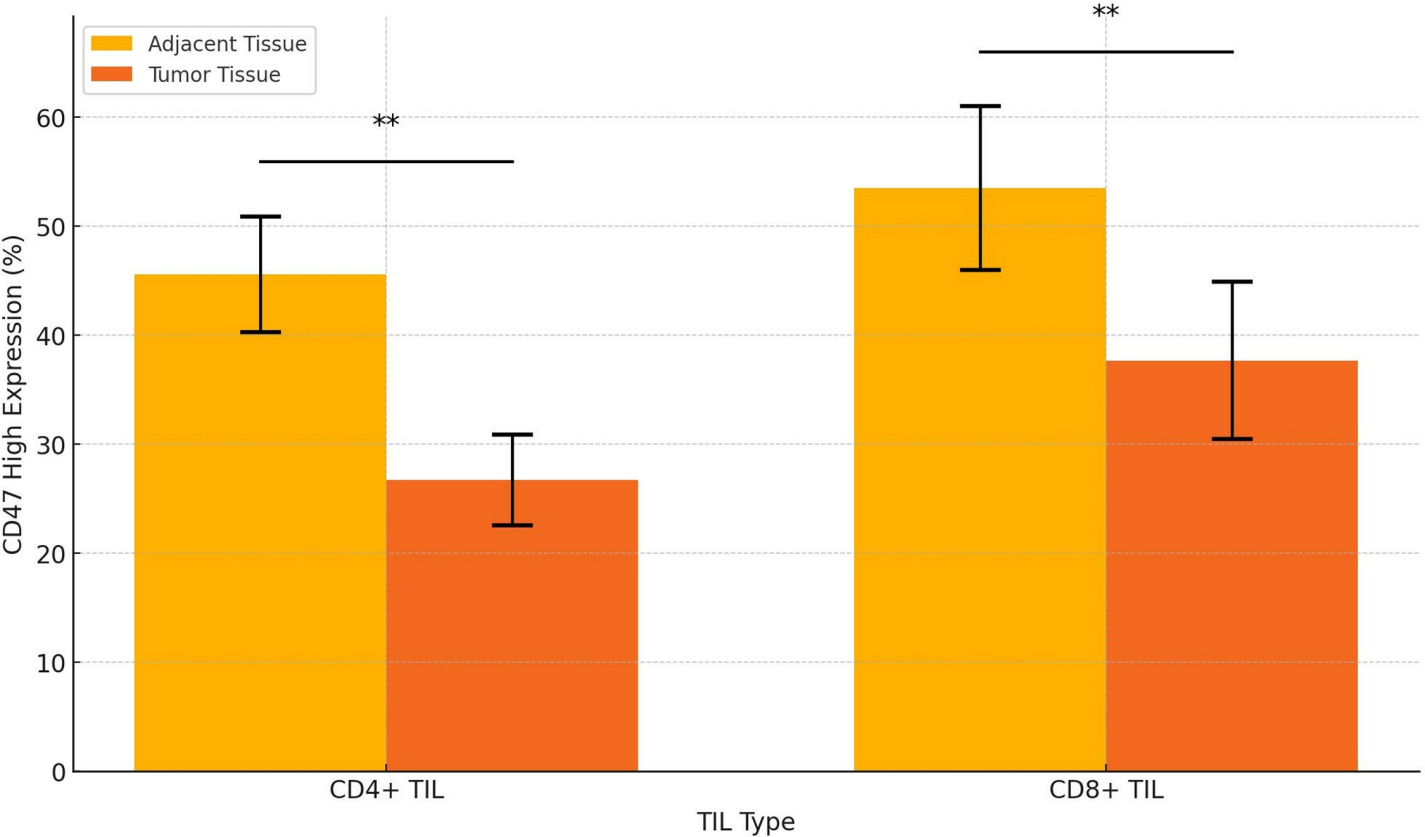
CD47 is lowly expressed on tumor-infiltrating CD4+ and CD8+ T lymphocytes in NSCLC.

In our study of NSCLC specimens, we assessed CD47 expression on tumor-infiltrating T lymphocytes using flow cytometry in tumor and adjacent normal tissues from 50 patients. Results showed high CD47 expression on 45.6% ± 5.31% of CD4+ and 53.5% ± 7.51% of CD8+ lymphocytes in adjacent tissues, whereas in tumor tissues, CD47high+ lymphocytes decreased to 26.7% ± 4.16% for CD4+ and 37.7% ± 7.21% for CD8+. Thus, CD47 expression is upregulated on tumor-infiltrating lymphocytes (Fig 8).

## 4. Discussion

In recent years, the incidence of lung cancer has remained high, with NSCLC being a common subtype, primarily represented by squamous cell carcinoma and adenocarcinoma [13–15]. Due to the lack of specific clinical symptoms, approximately 70% of NSCLC patients are diagnosed at an advanced stage, with poor prognosis overall [16]. The 5-year survival rate for advanced NSCLC patients is below 3% [17]. Standard chemotherapy and radiotherapy regimens are often limited in efficacy and safety due to patient tolerance, making the search for new therapeutic targets crucial for improving prognosis in NSCLC.

CD47, a cell surface protein in the immunoglobulin superfamily, is involved in various biological responses, including immune homeostasis and immune response generation [18,19]. CD47 is overexpressed to varying degrees in most tumor cells compared to adjacent tissues. Leary et al. [20] demonstrated that CD47 promotes the proliferation and metastasis of ovarian cancer cells, with generally poor prognosis in these patients. However, some studies have found no correlation between CD47 expression and poor prognosis in breast cancer [21], indicating mixed results. With continued research, CD47 overexpression has been observed in various tumor types, influencing tumor cell behavior through direct and indirect pathways. Notably, Isenberg et al. [22] found that CD47 deletion significantly alters immune cell behavior post-radiation, improving survival rates. Our study found that CD47 expression in NSCLC cancer cells was significantly higher than in adjacent normal tissues (P < 0.05), aligning with findings from Bang et al. [23,24]. CD47 expression was correlated with lymph node metastasis, clinical stage, and differentiation degree (P < 0.05), suggesting that as the disease progresses, CD47 expression increases. High CD47 expression may facilitate immune evasion, metastasis, and proliferation in NSCLC cells. Cox multivariate analysis revealed that CD47 expression and clinical staging are independent risk factors for prognosis, suggesting that reducing CD47 expression could enhance immune response, inhibit tumor cell proliferation, and potentially serve as a specific therapeutic approach for NSCLC.

CD47 also plays a role in protecting normal cells from immune attack, contributing to immune homeostasis [25–28]. Enhancing CD47-targeted therapies to focus specifically on NSCLC cells could reduce side effects, offering a new approach for personalized NSCLC treatment. While promising, CD47-targeted therapies require careful consideration of potential side effects, as inhibiting CD47 may cause immune suppression, impacting patient health [29]. Methods to reduce CD47 expression include small molecule inhibitors, antibody-drug conjugates, and gene-editing techniques like CRISPR-Cas9. Combining these with immunotherapy may further improve outcomes while minimizing adverse effects.

Tumor-associated macrophages (TAMs) primarily produced by macrophages, play a role in immune regulation. Prior studies suggest an inverse correlation between TAM infiltration and patient survival, indicating that lower TAM infiltration is associated with longer survival [30–32]. This study confirmed that TAM infiltration significantly impacts NSCLC patients’ overall survival (P < 0.05), with higher infiltration in CD47-positive patients (P < 0.05). This suggests that CD47 may influence TAM differentiation and certain biological functions within the NSCLC microenvironment. Our study, using PLA technology, found a significant interaction between CD47 and VEGFR, CD36, potentially impacting angiogenesis and cell metabolism within the tumor microenvironment, promoting tumor cell survival and proliferation. The literature also highlights CD47’s multifaceted role beyond its traditional “don’t eat me” signal, offering new perspectives for CD47 as a therapeutic target [33,34]. Future research should explore this molecular interaction, providing insights into CD47-targeted treatment strategies. Additionally, low CD47 expression on tumor-infiltrating CD4+ and CD8+ T lymphocytes may limit immune escape, allowing tumor cells to be more susceptible to T cell attack. Studies, including by Am J Pathol, confirm that CD47 deletion alters immune cell behavior in radiated tumors, enhancing survival. Our findings emphasize CD47’s immunoregulatory role, especially regarding T cell activity. Recent studies revealed CD47’s co-expression with IFT57 and its impact on cancer prognosis, suggesting dual targeting of CD47 and IFT57 may enhance efficacy and reduce CD47-targeted therapy’s side effects [35]. Genes like IFT57 and CRACD could serve as biomarkers, advancing precision treatment strategies. Further research should examine CD47’s potential as a biomarker.

In conclusion, CD47 expression is an independent risk factor for poor prognosis in NSCLC patients, correlating with poor outcomes and TAM infiltration. CD47 could be a valuable biomarker for NSCLC progression, prognosis prediction, and a potential therapeutic target.

## Supporting information

S1 Dataset This dataset contains the raw data used for statistical analysis in this study. (XLSX)
